# Precursor cells from Atlantic salmon (*Salmo salar*) visceral fat holds the plasticity to differentiate into the osteogenic lineage

**DOI:** 10.1242/bio.201411338

**Published:** 2015-05-06

**Authors:** Elisabeth Ytteborg, Marijana Todorcevic, Aleksei Krasnov, Harald Takle, Inger Øien Kristiansen, Bente Ruyter

**Affiliations:** Nofima AS, P.O. Box 5010, NO-1430 Ås, Norway

**Keywords:** *In vitro*, Osteoblast, Adipocytes

## Abstract

In order to study the potential plasticity of Atlantic salmon (*Salmo salar*) precursor cells (aSPCs) from the adipogenic mesenchyme cell lineage to differentiate to the osteogenic lineage, aSPCs were isolated and cultivated under either osteogenic or adipogenic promoting conditions. The results strengthen the hypothesis that aSPCs most likely are predestined to the adipogenic lineage, but they also hold the flexibility to turn into other lineages given the right stimuli. This assumption is supported by the fact that the transcription factor *pparγ* , important for regulation of adiopogenesis, was silent in aSPCs grown in osteogenic media, while *runx2,* important for osteogenic differentiation, was not expressed in aSPCs cultivated in adipogenic media. After 2 weeks in osteogenic promoting conditions the cells started to deposit extracellular matrix and after 4 weeks, the cells started mineralizing secreted matrix. Microarray analyses revealed large-scale transcriptome responses to osteogenic medium after 2 days, changes remained stable at day 15 and decreased by magnitude at day 30. Induction was observed in many genes involved in osteogenic differentiation, growth factors, regulators of development, transporters and production of extracellular matrix. Transcriptome profile in differentiating adipocytes was markedly different from differentiating osteoblasts with far fewer genes changing activity. The number of regulated genes slowly increased at the mature stage, when adipocytes increased in size and accumulated lipids. This is the first report on *in vitro* differentiation of aSPCs from Atlantic salmon to mineralizing osteogenic cells. This cell model system provides a new valuable tool for studying osteoblastogenesis in fish.

## INTRODUCTION

Mesenchymal stem cells (MSCs) have the capacity to differentiate into a number of cell types, including osteoblasts, chondroblasts, adipocytes and myocytes (Mizuno, 2009; Pittenger et al., 1999; Pittenger et al., 2000). The pluripotency of these cells is reflected by the large quantity of regulatory factors controlling their fate. The regulation of the commitment to differentiate to several cell lineages is currently an area of intense research interest. Fat is an ideal tissue to acquire MSCs (Zuk et al., 2001), since a relatively large quantity of fat is present in the Atlantic salmon (*Salmo salar*) abdominal cavity. We hypothesized that these cells from Atlantic salmon can be stimulated to differentiate into both the adipogenic and osteogenic lineages *in vitro*, as previously demonstrated for human subcutaneous fat derived stem cells (Zuk et al., 2001).

The microenvironment of stem cells plays an important role in cell behavior, function and pre-determination of cell lineage fate (Arai et al., 2004). Adipogenic and osteogenic cells share many common features at early stages of development prior to their commitment to a certain cell lineage, followed by more different features at the later stages after commitment to a specific lineage. The differentiation into adipogenic or osteogenic lineage is controlled by activation or silencing of different genes, signaling molecules and transcription factors. Therefore, specific markers are required to confirm whether precursor cells have successfully matured into a different type of specialised cell. Osteoblasts express several phenotypic markers, such as collagenous (e.g. Collagen1a) and non-collagenous (e.g. Osteocalcin, Osteonectin) bone matrix proteins (Aubin, 1998), controlled by a suite of signaling molecules (e.g. bone morphogenetic proteins, BMPs) and transcription factors (e.g. Runx2 and Osterix) (Komori, 2005; Lee et al., 2003; Nishio et al., 2006; Aubin, 1998; Franz-Odendaal et al., 2006). Differentiation of adipocytes is initiated through a set of signals, where the transcription factor C/EBP plays an important role by activating transcription of the nuclear receptor PPARγ, which then controls the differentiation programs of MSCs into adipogenesis instead of osteogenesis (Giaginis et al., 2007a; Giaginis et al., 2007b). Cellular determination and differentiation pathways have been little studied in teleosts, and most knowledge is obtained from the mammalian research. Recent gene expression studies in Atlantic salmon demonstrated that the process of osteoblastogenesis and adipogenesis is overall similar to that in mammals (Ytteborg et al., 2013; Ytteborg et al., 2010a; Todorčević et al., 2010b; Todorčević et al., 2010a). However, differences in bone formation do exist (Ytteborg et al., 2013; Witten and Huysseune, 2009), making Atlantic salmon an interesting organism for comparative studies of cellular differentiation and development.

It is important to understand not only normal regulation of cell fate decisions, but also mis-regulation of mesenchymal differentiation that results in development of disorders. Trans-differentiation and cellular determination has been suggested to be involved in the disease states, such as vertebral malformations in salmon (Ytteborg et al., 2010a,b,c; Helland et al., 2006), and in healing processes, for example distraction of osteogenesis in rats (Yasui et al., 1997; Choi et al., 2002). There is a clinical correlation between the appearance of bone marrow fat and reduced bone forming capacity in human patients (Gimble and Nuttall, 2012). It has been suggested that inhibition of adipogenesis in the bone marrow may stimulate osteogenesis (Nuttall et al., 2014). Clinical studies have shown that bone loss in osteoporotic patients and in people with age-dependent problems is associated with increased adipose tissue in the bone marrow (Nuttall and Gimble, 2004). Similar pathological conditions exist in farmed fish including Atlantic salmon, where hyper-mineralization, trans-differentiation of chondrocytes into osteoblasts and adipocytes into chondrocytes, increased bone formation and/or heterotopic bone formation in the vertebral bodies occur (Ytteborg et al., 2010a; Helland et al., 2006; Kranenbarg et al., 2005). Trans-differentiation of cells takes place in particular cases, for example transformation of adipose tissue into cartilage during development of hyper dense vertebrae in response to phytic acid in salmon (Helland et al., 2006), and osteoblasts into adipocytes in humans during diabetes and aging (Moerman et al., 2004). Impact of nutrition, growth, temperature and other conditions on osteogenic differentiation and bone formation are of great interest and importance for the aquaculture industry.

Three models of osteoblast-like cell cultures derived from fish tissues have so far been described; two cell lines (Pombinho et al., 2004) and two primary cultures (Capilla et al., 2011) from sea bream (*Sparus aurata*) calcified tissue and one derived from salmon white muscle precursor cells (Ytteborg et al., 2010b). However, osteoblasts derived from Atlantic salmon that are capable of mineralizing *in vitro* have not been described. We hypothesis that precursor cells isolated from the visceral fat of Atlantic salmon (aSPCs) are predestined to the adipogenic lineage, but that they also hold the flexibility to turn into other lineages given the right stimuli. In the presented study, we established a cell culture system where aSPCs differentiate into lipid accumulating adipocytes (aSACs) and mineralizing osteoblasts (aSOCs). Using lineage specific molecular markers, microarray and morphological observations, we characterized the differentiation process of aSACs and aSOCs from the common MSC origin aSPCs.

## RESULTS

### Cell morphology

**Fig. 1.**
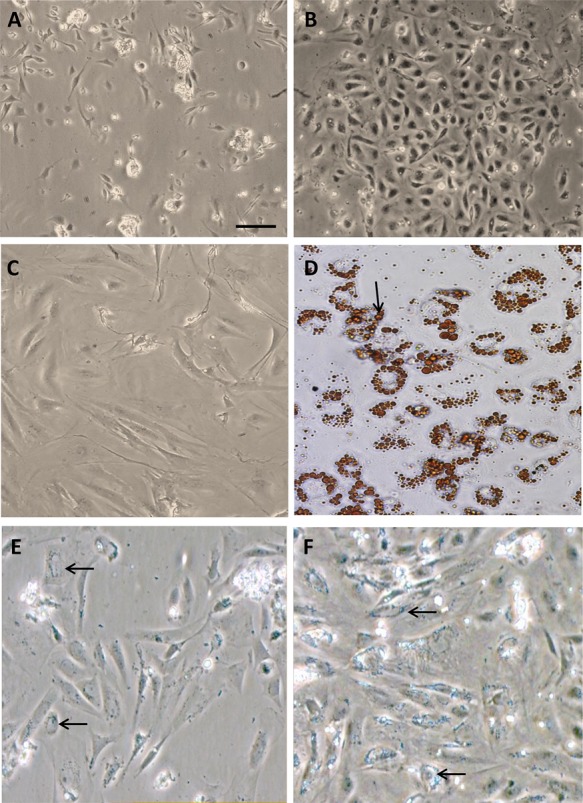
**Newly islated aSPCs and on adipogenic differentiation (DMEM-AD) media.** (A) Newly isolated aSPCs. (B) Confluent cells prior to differentiation media. (C) Cells given adipogenic differentiation media (DMEM-AD) day 15, notice the different and enlarged shape. (D) Oil Red O stained aSACs after 30 days. Arrow indicates accumulation of lipids within each cell. (E,F) aSPCs left in growth media for 15 days also started accumulating lipids (as indicated by arrows). In some areas, these cells detached from the wells leaving few cells left (E) whereas other regions remained dense (F). Scale bar=10 µm.

**Fig. 2.**
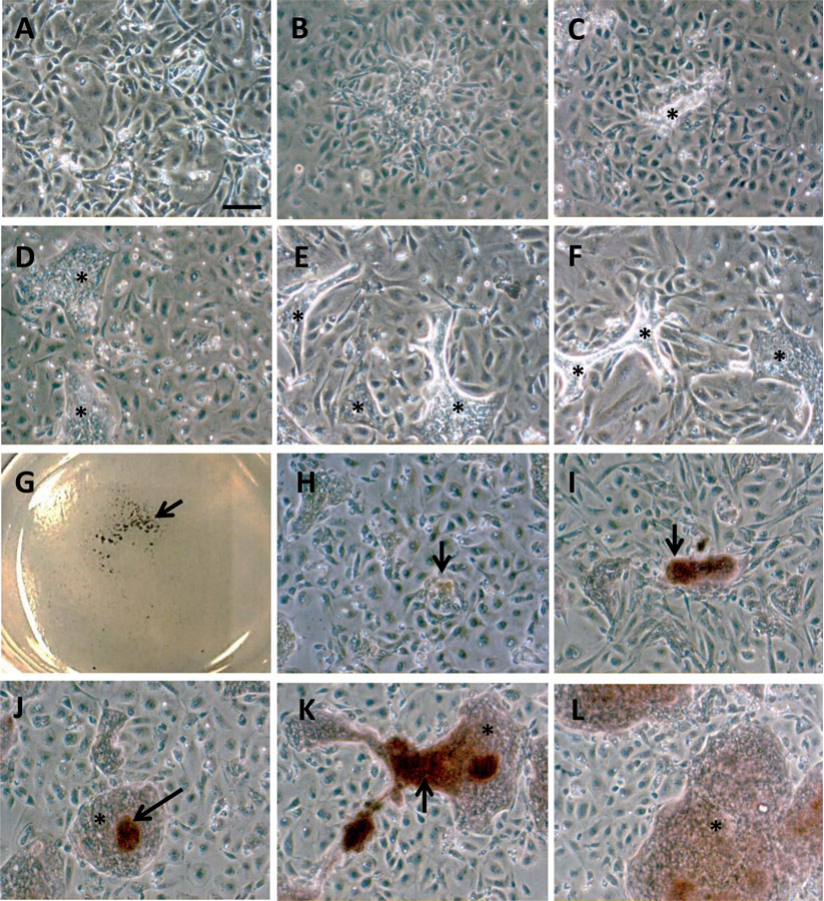
**Cells on osteogenic differentiation (DMEM-OB) media.** (A) 2 days after adding DMEM-OB. (B) 7 days after added DMEM-OB. (C) 15 days after adding DMEM-OB, notice the beginning of mineralization and mineralizing nodules. (D-F) 30 days after adding DMEM-OB. Notice large regions of mineralized matrix. (G) Alizarin staining of aSOCs, image of well with cells. Arrow indicates dark staining of mineralized nodules. (H) Beginning of mineralization. (I) Mineralized region in the middle of clustering cells. (J,K) Matrix was more mineralized in regions were matrix was dense. (L) Large mineralized regions. Asterisk indicates matrix, arrows indicate intensive alizarin stained matrix. Scale bar=10 µm.

Newly isolated aSPCs were small with a cytoplasm devoid of lipid droplets and morphologically very similar to fibroblasts (Fig. 1A). They had a high proliferation capacity and reached confluence seven days after seeding (Fig. 1B). Cells cultivated in adipogenic media increased in size during the differentiation period (Fig. 1C), before they adopted the typical rounded shape of adipocytes containing intracellular lipid droplets (Fig. 1D), as previously described (Todorčević et al., 2010a). At day 30 the cytoplasm was filled with lipid droplets, shown by staining cells with Oil Red O (Fig. 1D). aSPCs that were kept on growth media, with no additional lipids except those from FBS, started accumulating lipids like the adipocytes after 15 days (Fig. 1E,F), however not to the same extent as cells given adipogenic media containing high concentrations of lipids. Cells grown in osteoblast promoting media gradually changed their morphology from a rounded shape to a more compact, cobblestoned shape (Fig. 2A), as previously described (Capilla et al., 2011; Ytteborg et al., 2010d). Regions where mineralized nodules appeared were visible after 15 days (Fig. 2B). In these regions, cells formed denser clusters where matrix gradually was deposited (Fig. 2C) and seen as bright fields in the microscopy (Fig. 2C-F). After 30 days in cultures larger regions with matrix were observed (Fig. 2E,F). Staining with Alizarin Red S showed mineralization of the secreted matrix (Fig. 2G-I). Only weak staining was observed after 15 days (Fig. 2H). After 30 days, grade of mineralization varied in the secreted matrix. In some regions with limited deposition of matrix intense staining was observed (Fig. 2I). Weak staining was seen along the rims of the secreted matrix in other regions (Fig. 2J). In regions with more matrix deposited, both strong (Fig. 2K) and weak (Fig. 2L) staining was observed. Heterogeneous staining pattern was observed in the same well.

### Gene markers of osteoblast and adipocyte differentiation

**Fig. 3.**
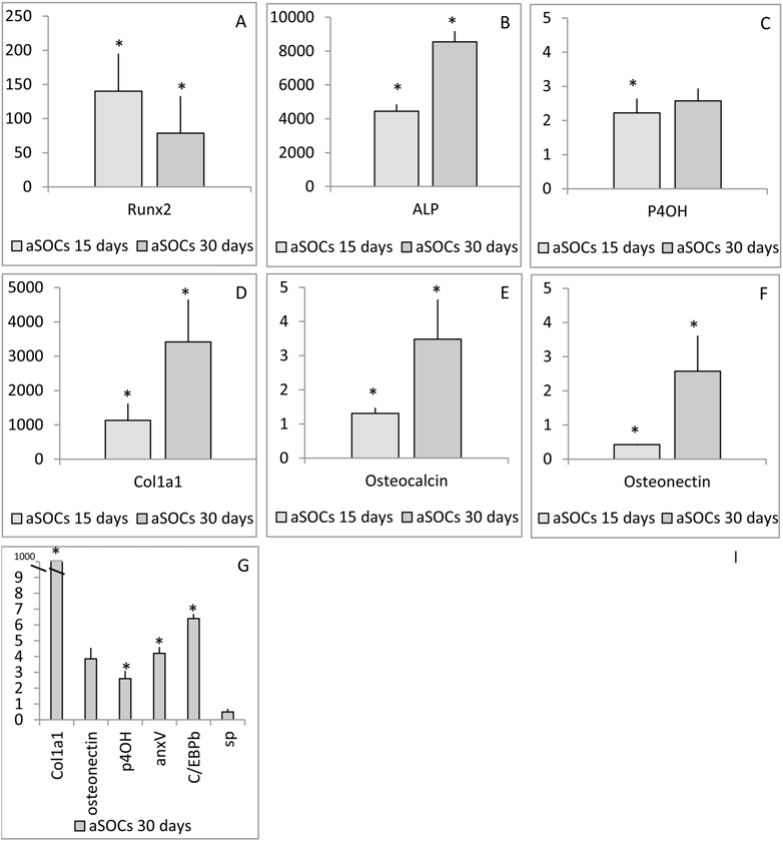
**Real time qPCR of osteogenic and adipogenic gene markers.** (A) *runx2*, (B) *alp*, (C) *p4oh*, (D) *col1a1*, (E) *osteocalcin* and (F) *osteonectin* transcription in aSOCs at day 15 and 30 compared to aSPCs. (G) *col1a1*, *osteonectin*, *p4oh*, *anxV* and *C/EBPβ* transcription in aSOCs at day 30 compared to aSACs at day 30. Data are given as mean values, error bars show SE, n=4 and significant differences (P=0.05) are indicated by *.

We analyzed transcription of osteoblast and adipocyte specific genes in aSOCs and aSACs with qPCR at day 7, 9, 15 and 30 (Fig. 3A-G). Gene expression results confirmed that the cells were entering different lineages: *pparγ* mRNA was absent in cultures given osteogenic media and *runx2* transcription was not detected in cultures given adipogenic media at day 9 (results not shown). The transcription of *runx2* decreased at day 30, when the cells had high matrix deposition. Simultaneously an increase of *alkaline phosphatase (alp)*, *prolyl 4 hydroxylase (p4oh), collagen 1a1 (col1a1), osteocalcin,* and *osteonectin* mRNA levels was observed at day 30. In addition, *bmp4* was detected at day 15 and *annexin V (anxV)* at day 30, however, these two genes were transcribed at very low levels at other stages and relative transcription could not be calculated (results not shown). Compared to aSACs, expression of *col1a1, osteonectin* (ns)*, p4oh, anxV* and *c/ebpb* was higher in aSOCs, while level of *substance P* (*sp*) was lower, albeit insignificantly (Fig. 3G).

### Microarray

**Fig. 4.**
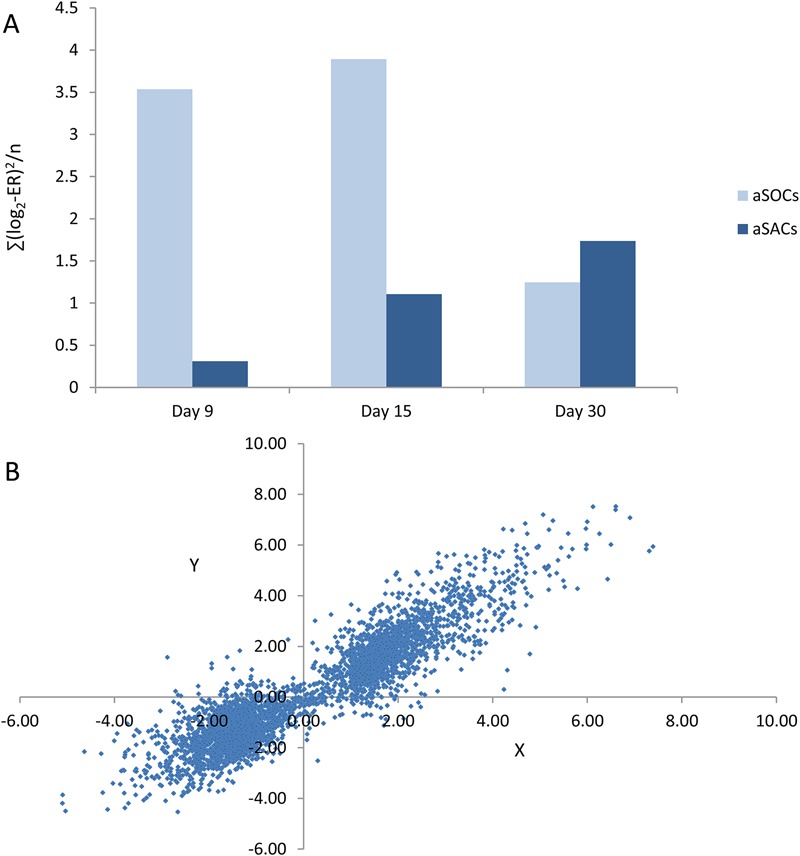
**An overview of transcriptome changes during differentiation of aSOCs and aSACs: summary of microarray analyses.** (A) Magnitude of transcriptome changes estimated as ∑(log_2_-Expression Ratio)^2^/n (n = number of genes) was different between the cell cultures and time-points. (B) Close concordance of transcription profiles in aSOCs at days 9 and 15. Each dot corresponds to a gene and presents log_2_-Expression Ratios at two time-points: day 9 (X-axis) and day 15 (Y-axis).

Targeted qPCR analyses were supplemented with transcriptome profiling. Both magnitude and time-course of gene expression changes were markedly different in the two cell lineages. Large scale response to the osteogenic differentiation medium was seen already after two days, at day 9, with similar findings at day 15, but a decrease in magnitude was observed at the end of the experiment, day 30 (Fig. 4A). The transcriptome response in aSACs was initially smaller, but increased with time and eventually was slightly higher than in aSOCs at day 30 (Fig. 4B). Expression profiles of aSOCs at day 9 and 15 were tightly correlated (Pearson r=0.95).

**Fig. 5.**
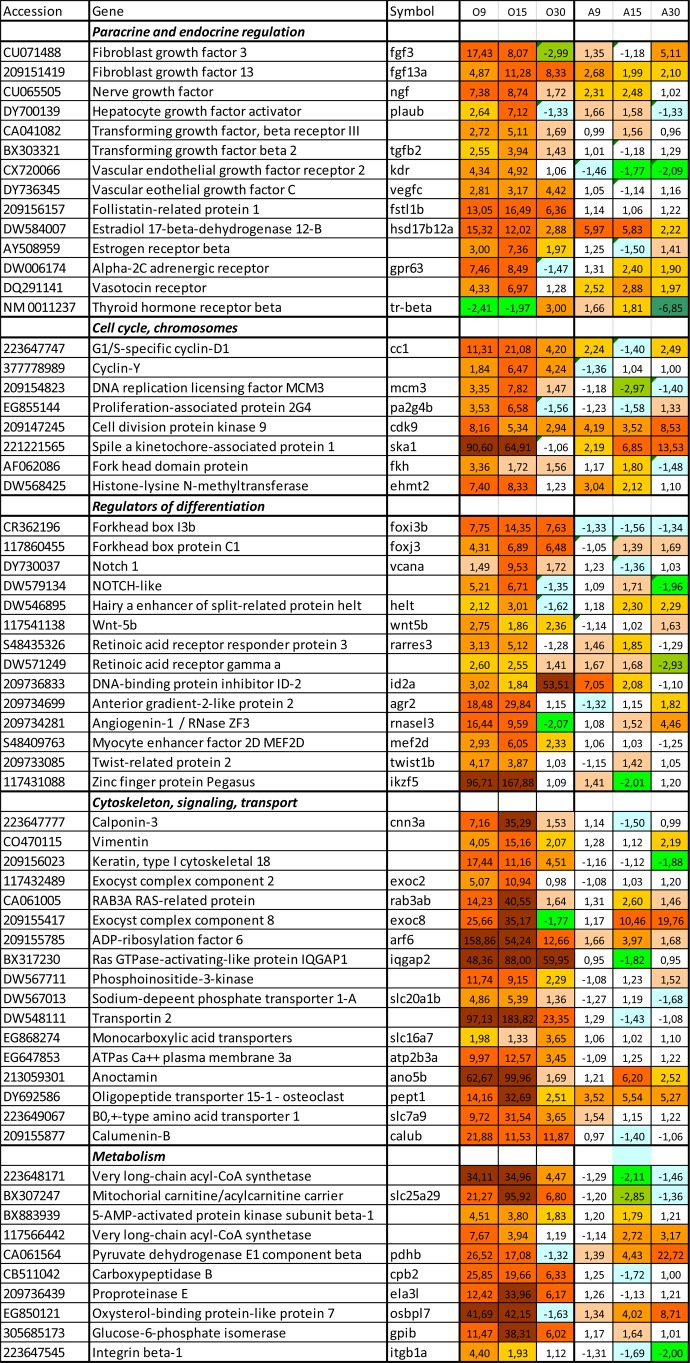
**Differentiation of aSOCs was associated with expression changes of genes involved in paracrine and endocrine regulation, cell cycle and control of differentiation.** Genes encoding components of cytoskeleton and proteins involved in transport and metabolism were also induced. Part of these changes were common for both cell types, however multiple genes were regulated only in aSOCs. Microarray analyses, data are folds to control (day 7).

**Fig. 6.**
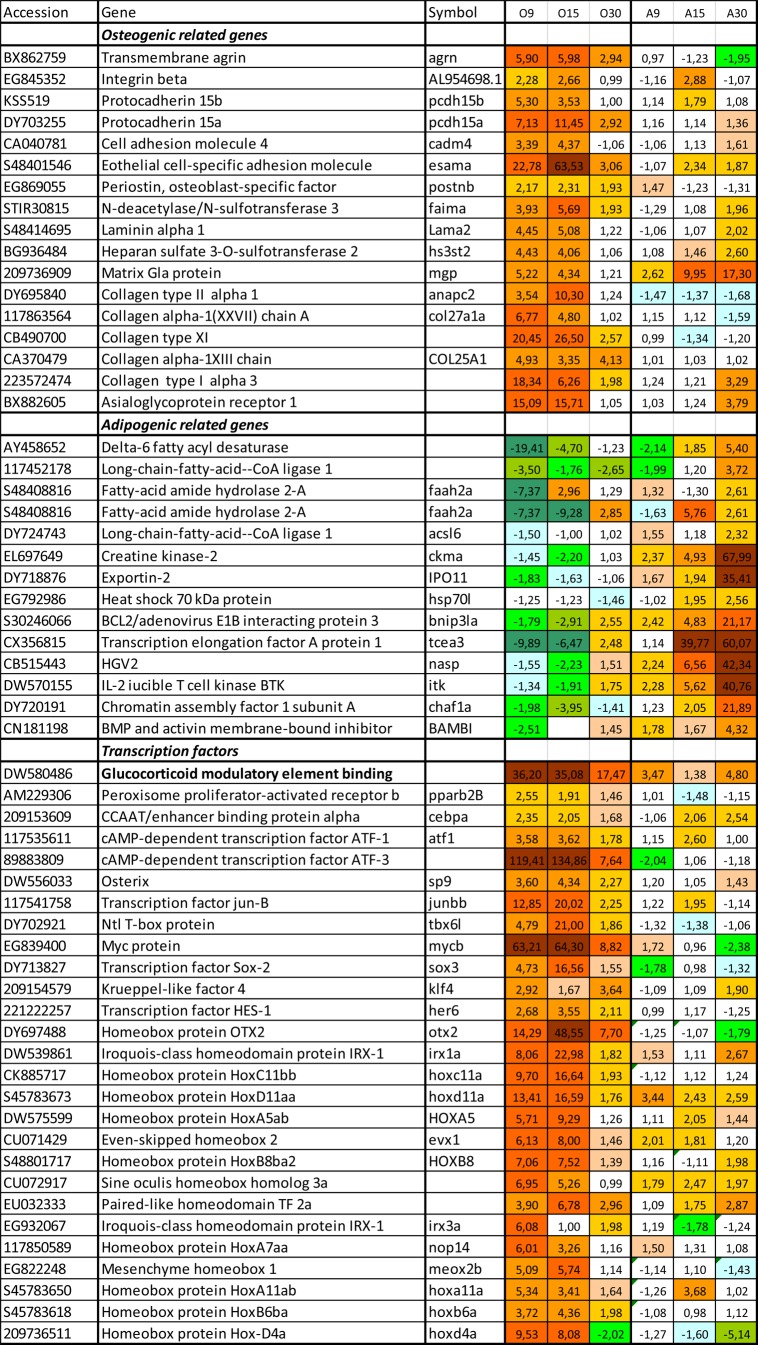
**Differentiation of aSOCs was in line with up-regulation of multiple transcription factors and genes encoding proteins involved in cell adhesion and formation of extracellular matrix.** Preferential up-regulation in aSACs was observed in a suite of genes encoding enzymes of lipid and energy metabolism and proteins with unknown roles in adipocytes. Microarray analyses, data are folds to control (day 7).

Differential expression was shown by multiple genes involved in signaling and regulation of differentiation. Increased expression of growth factors and receptors to growth factors and hormones (Fig. 5) indicated paracrine and endocrine control of aSOC differentiation. Up-regulation *hsd17b12a* and *estrogen receptor* was in line with the role of sex hormones in osteogenesis. *Receptor to thyroid hormone (tr-beta)*, which plays an important role in calcium homeostasis, was up-regulated at day 30. Thyroid receptors (TRs) have been demonstrated in rat, mouse and human osteoblasts (Sato et al., 1987; LeBron et al., 1989; Allain et al., 1996). Several growth factors have unknown roles in fish. The osteoblast medium activated a suite of genes involved in regulation of the early stages of embryonic development when patterning and speciation of organs, tissues and cell types takes place; these include components of Notch, Wnt and retinoic acids pathways, pathways that are involved in osteogenesis in mammals (Kobayashi and Kronenberg, 2005). Mesenchymal development requires commitment of cells to restricted lineages, which includes epigenetic regulation of chromatin remodeling (Lee et al., 2004; Illi et al., 2005). Several genes with important roles in histone modifications, including *hdac3, fkh* and *ehmt2,* showed higher expression in aSOCs compared to aSACs. Highly induced *agr2* controls production of extracellular proteins (Salmans et al., 2013). Both positive and negative (*foxj3, id2a, fstl1b* and *twist1b*) regulators of differentiation (Karsenty, 2008) had increased expression, indicating highly complex and controlled mechanisms in the differentiation processes. For example *id2a*, which counter acts multiple HLH transcription factors (TF) was one of most up-regulated genes at day 30 (Fig. 6). A large group of activated genes encode TF, many of which control differentiation of diverse cell types (e.g. *ppar2b, cebpa, sox3, junbb, hes1, klf4*). Several TFs are known to be involved in osteoblast differentiation. *Sp9* or *osterix* is a runx2 activated TF essential for bone formation (Komori, 2005; Lee et al., 2003; Nishio et al., 2006; Aubin, 1998; Franz-Odendaal et al., 2006). *Glucocorticoid modulatory element binding protein 2* that showed high expression changes within the whole study period is also involved in osteogenic differentiation of MSCs (Olkku and Mahonen, 2008). The cAMP-dependent TF binding to the response element (CRE) are known to regulate endochondral bone development (Pearman et al., 1996). A large group of TF contain homeobox domains (hox); this structural feature is common for multiple regulators of embryonic development and differentiation. Many genes encoded putative TFs containing zinc finger domains with unknown roles (data not shown).

Enhanced proliferation was mirrored by gene expression profiles. Greatest changes were shown by *cc1*, one of the key regulators of cell cycle and *ska1* involved in spindle formation during mitosis (Wang et al., 2010; Sun et al., 2014). The observed changes in aSOCs phenotype were associated with induction of multiple genes for proteins of cytoskeleton, transporters and enzymes. *Vimentin* and *keratin 18* are specific for respectively mesenchymal and epithelial cells (Kalluri and Weinberg, 2009; Abe and Oshima, 1990). Strong induction (up to 160-fold) was seen in a suite of proteins involved in exocytosis (*exoc2, rab3ab, exoc8, arf6* and *iqgap2*). Osteogenic medium also induced several genes coding for transporter proteins, including *slc20a1b*, *ano5b* and *atp2b3a*, which transfer calcium and phosphate and also *osteoclast-specific pept1* (Zoidis et al., 2004; Yoshiko et al., 2003). *Calumenin B* regulates vitamin K-dependent carboxylation of multiple N-terminal glutamate residues and binds calcium ions with low affinity; this gene was highly induced during the whole differentiation period, being markedly down-regulated in adipocytes. Bone formation presumes establishment of tight contacts between cells and formation of extracellular matrix. Up-regulation was shown by several genes involved in cell contacts and adhesion and similar transcription changes were displayed by genes encoding collagens of different types. For example, *postnb*, an osteoblast-specific protein with an important role in cell-collagen interactions and *mgp*, an extracellular calcium binding protein that participates in ossification. Increased expression was shown by genes encoding proteins of tight junctions that are important for osteoblast maturation and regulation of ion transport across the bone membrane (Wongdee et al., 2008). A small number of genes including five enzymes of lipid metabolism were up-regulated exclusively in adipocytes. Strongest regulation in the aSACs was observed in genes whose role in this cell type is not well known. Interestingly, several enzymes of lipid metabolism showed greater up-regulation in aSOCs than in aSACs.

## DISCUSSION

This study demonstrated for the first time that Atlantic salmon precursor cells from the adipogenic mesenchyme cell lineage may differentiate to mineralizing, osteoblast-like cells *in vitro* and that salmon MSCs isolated from adipose tissue holds the flexibility to differentiate into various cell lineages. Mammalian MSCs are pluripotent cells that give rise to diverse mesodermal cell types including osteoblasts, adipocytes, chondrocytes and myocytes, both *in vitro* and *in vivo* (Mizuno, 2009; Pittenger et al., 1999; Pittenger, 2013; Abdallah and Kassem, 2008). A number of studies have focused on the inter-conversion between mammalian adipocytes and osteoblasts. Single MSC-derived clones have been shown to have an ability to differentiate into adipocytes, dedifferentiate, and subsequently differentiate to osteoblasts *in vitro* (Song and Tuan, 2004). Mature osteoblasts are also able to undergo adipogenesis (Nuttall et al., 1998) and mature adipocytes can be redirected towards an osteoblast pathway by manipulation with culture conditions (Justesen et al., 2004). Trans-differentiation of mammalian bone marrow adipocytes (Song and Tuan, 2004) and subcutaneous pre-adipocytes (Justesen et al., 2004) into osteoblasts has been reported. Similarly to mammalian MSCs, aSPCs from Atlantic salmon showed high plasticity in our experiments. When exposed to osteogenic media, aSPCs acquired a number of features specific for osteoblasts. A hallmark was their ability to secrete and mineralize ECM through deposition of calcium phosphate *in vitro*.

Differentiation of aSPCs into aSOCs was characterized with loss of adipogenic (*pparγ*) and increase of osteogenic (*runx2, osterix*) mRNA transcription. This indicated that the precursor cells isolated from adipose tissue were pre-determined to the adipogenic lineage. This was further confirmed in aSPCs given only growth media, where the aSPCs started to accumulate lipids in their cytoplasm without being subjected to adipogenic differentiation media. Inactivation of adipogenic genes indicated that precursor cells isolated from adipose tissue differentiated into another lineage upon external cues. Osteogenic markers, such as *alp* and *osteocalcin* showed increased transcription in aSOCs, supporting the morphological observations that they were entering the osteogenic lineage. Somewhat unexpectedly, c*/ebpβ*, an important transcription factor in regulation of adipogenesis had increased mRNA expression in aSOCs. However, the gene product of c*/ebpβ* is also documented as a trans-activator of *osteocalcin* and *col1a* (Tominaga et al., 2008), hence suggesting that a combinatorial interaction of these factors regulates tissue-specific transcription during osteoblast differentiation. Importantly, *col1a1* mRNA expression was up-regulated during differentiation in aSOCs, but down-regulated in aSACs and the transcription further increased from day 15 to day 30 in aSOCs. This corresponded with increased transcription of other matrix producing genes (*osteonectin, osteocalcin, alp* and *p4oh*). Along with increased transcription of matrix producing genes, increased matrix secretion was observed in the cultures. All in all, specific gene expression profile along with microscopic examination of cellular morphology and secreted matrix strongly suggests that the aSPCs differentiated into cells belonging to the osteoblast lineage.

Microarray analyses brought additional evidence to a view that the aSPCs were pre-committed to adipogenic differentiation. While addition of osteogenic medium produced a dramatic perturbation of the developmental program, magnitude of transcriptomic responses to adipogenic stimulation was small and gradually increased to the end of the experiment as might be expected for committed cells. Change of trajectory presumes suppression of pre-adipocyte phenotype and reconfiguration of the entire program. Judging by timing, number of differentially expressed genes and magnitude of changes, the decision seems to be taken promptly (day 9) with a cost that was quite high. Induction was shown by a number of genes involved in the whole range of processes related to cell communication and proliferation, differentiation and metabolism, formation of cytoskeleton, cellular layers and extracellular matrix. High correlation of expression profiles at days 9 and 15 suggest consistency of changes. It is noteworthy, that major part of responses was transient and their manifestation ceased after visible signs of osteoblast maturation appeared as mineralized nodules in the culture at day 15. Striking magnitude and complexity of transcriptome responses could be partly accounted for by heterogeneity of the primary aSPCs culture: it might include diverse cell types that developed different reactions to the osteogenic medium. A complementary view is that reprogramming of differentiation switched on a search mode and sorting of options. As seen in the end of the experiment, maintenance of novel phenotype required a relatively small number of up-regulated genes and in this respect aSOC and aSAC were similar.

Overall, our results confirmed that the primary culture established in the presented work belongs to cells that qualify as osteoblasts. In the aquaculture industry, numerous problems regarding bone development and mineralization are detected in response to nutritional and environmental challenges. Thus, the established *in vitro* system can be a promising model for studies of osteoblastogenesis in fish.

## MATERIALS AND METHODS

### Cell isolation

The aSPCs were isolated from Atlantic salmon with an average weight of 500 g. Fish were sedated (Tricaine methane sulphate, Pharmaq, Norway) and visceral fat was harvested and collected in L-15 medium supplemented with 10 ml/l antibiotic/antimycotic (Sigma-Aldrich, St. Louis, MO USA). Tissue was minced and centrifuged at 250 ***g*** for 15 min and washed twice with L-15 before digestion with 0,125% collagenase for 60 min at 11°C. Cells were filtered through 250 and 100 µm filters and washed twice in L-15 and once in DMEM-growth media (DMEM-GM) (Table 1). Cells were then separated using gradient centrifugation; 1 part 1.5 M NaCl and 9 parts of Percoll solution (Sigma, MI USA) were mixed and diluted to 36% with L-15. Using tubes (p/n326823, Beckman Coulter, Inc., CA USA) filled with 30 ml 36% solution in each tube were centrifuged at 60 000 ***g*** (SW 28, Beckman Coulter) for 17 min. Cell suspension (2 ml) was gently put on top of the Percoll gradient and centrifuged at 1000 ***g*** for 27 min. The upper layer contains the mature cells, while the smaller stem cells reside in the middle layer. The middle layer was collected, centrifuged at 500 ***g*** for 10 min, supernatant was removed and pellet was dissolved in growth media. Cells were then seeded out and placed in an incubator, 12°C with 5% CO_2_. Medium was changed first after 24 h and then every 2–3 days. Cells were grown in triplicate cultures for each time-point on growth media until confluence, and the experiment was repeated 3 times. After reaching confluence, cells were subjected to either osteogenic differentiation media (DMEM-OB) (Table 1) or adipogenic media (DMEM-AD) (Table 1) for 30 days as described previously (Ytteborg et al., 2010d; Todorčević et al., 2008). Differentiation was carried out at 12°C and 5% CO_2_ and the media were changed every 3 days.

### RNA isolation and cDNA synthesis

Cells were harvested at day 7 (confluence), day 9 (2 days after addition of differentiation media), day 15 (one week in differentiation media) and day 30 (3 weeks after adding differentiation media). Cells were washed twice with PBS and harvested in DTTT and RLT buffer (Qiagen, Hilden, Germany). Total RNA was isolated using an RNeasy® Mini Kit and QIAshredder columns with on-column RNase-Free DNase set (Qiagen), all in accordance with the manufacturer's protocols. The total RNA concentration and quality were determined by spectrophotometry (NanoDrop® ND-1000 Spectrophotometer, NanoDrop Technologies, Wilmington, DE USA). 1 µg of total RNA was reverse transcribed to cDNA in a total volume of 50 µl using an oligo(dT) primer and reagents from the TaqMan Gold RT-PCR kit (Applied Biosystems, CA USA).

**Table 1.**
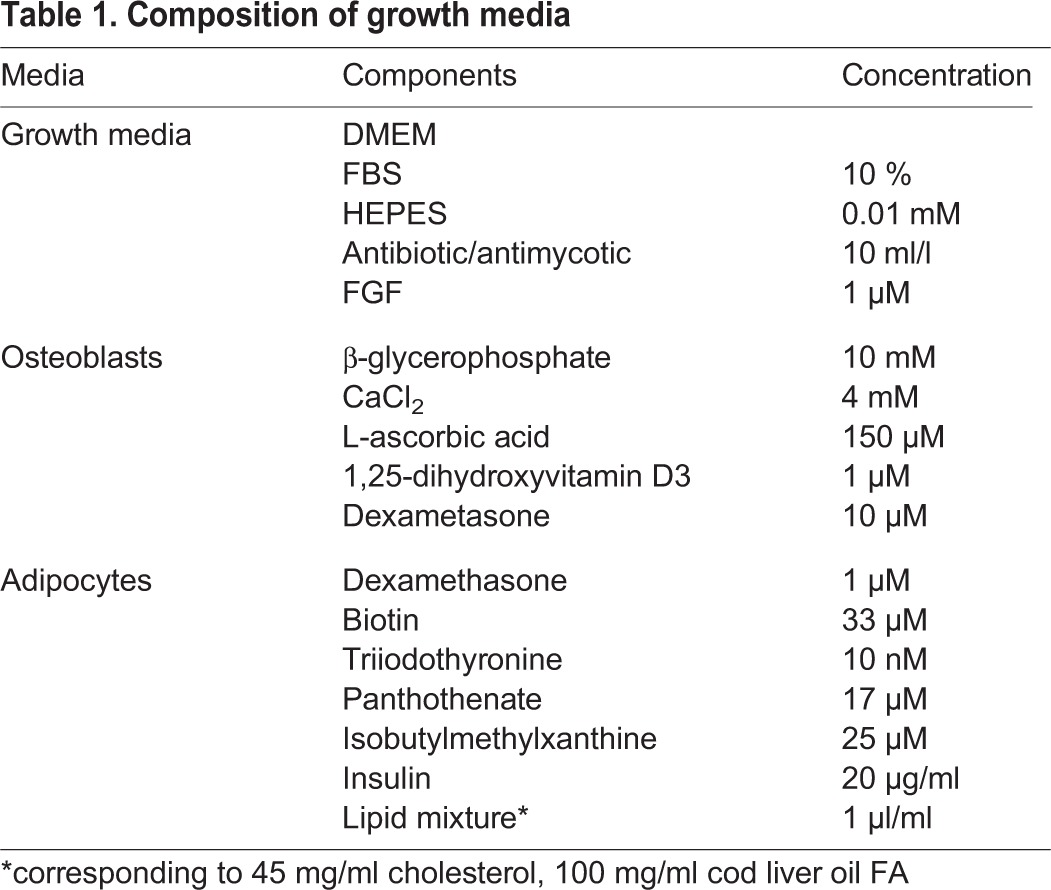
**Composition of growth media**

### Real–time qPCR

Primers (Table 2) were designed using the Vector NTI Advance 10 (Invitrogen) and NetPrimer (PREMIER Biosoft, CA, USA) software. PCR products were inserted into pGEM T-easy vectors (Promega, WI, USA), sequenced in both directions and their identity was verified with BLAST. Fluorescence-based real-time qPCR was performed using the Lightcycler LC480 (Roche). The reactions were run through the following programme: 95°C for 10 min, followed by 40 cycles at 95°C for 15 sec and 60°C for 1 min. Further, specificity was assessed by the melting curves (95°C for 15 sec, 60°C for 1 min, and 97°C continuous). PCR efficiencies were determined for each assay, ef1a used as a reference and transcription ratios calculated by the Relative Expression Software Tool (REST) (Pfaffl et al., 2002).

**Table 2.**
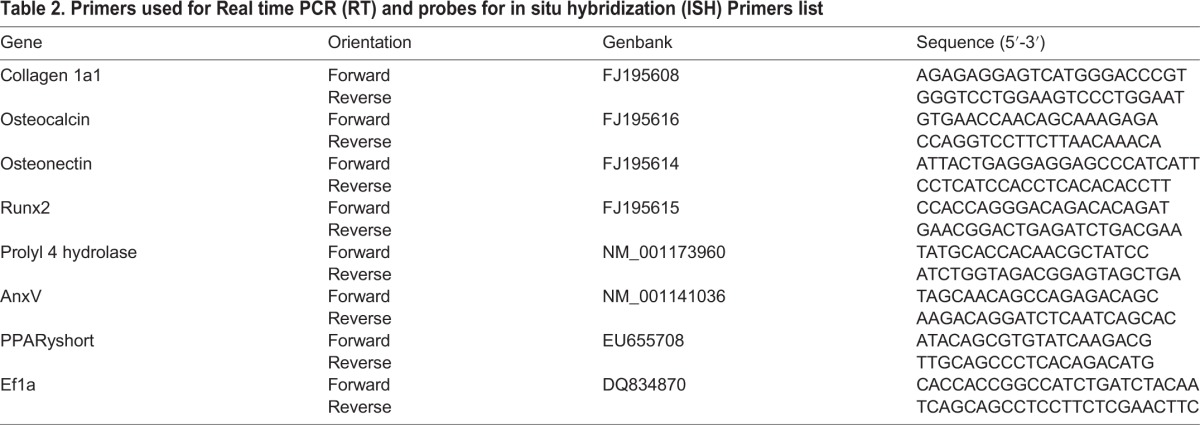
**Primers used for Real time PCR (RT) and probes for in situ hybridization (ISH) Primers list**

### Staining

Differentiation of aSPCs into aSOCs was evaluated by analyzing the deposition of minerals in the extracellular matrix detected by Alizarin Red S staining in cells growing in control or mineralizing media. Medium was carefully aspirated from each well, washed twice with PBS and fixated in ice cold 70% ethanol for 1 hour at room temperature. Alcohol was removed and wells rinsed twice with PBS. 2% Alizarin Red S, pH 4.1–4.3, was added so that it covered the well and incubated at room temperature for 15 min with gentle shaking. Alizarin Red S was removed and wells washed four times with dH_2_O. Oil Red O was used to visualize lipid droplets in aSACs. Briefly, cells were fixated as described above. Oil Red O was added to the wells so that it covered the cells completely for 5 min. Oil Red was removed after 5 min and wells were washed in 60% isopropanol before the nuclei were stained with haematoxylin for 2 min. Cells were rinsed with water, glycerol added and cells microscopically analysed (Leica Biosystems).

### Microarray

Gene expression profiling was carried out at days 7, 9, 15 and 30. Equal inputs from five aSPCs, aSACs and aSOCs cultures were pooled in each sample; three replicates per culture and time-point were analyzed. Equalized control was prepared by mixing RNA from all samples. Nofima's Atlantic salmon oligonucleotide microarray (GPL10705) and bioinformatic system STARS were used. The platform includes 21,000 unique probes spotted in duplicate. Microarrays were manufactured by Agilent Technologies (Santa Clara, CA USA) and unless indicated otherwise, the reagents and equipment were from the same source. RNA amplification and labeling were performed with a Two-Colour Quick Amp Labelling Kit and a Gene Expression Hybridization kit was used for fragmentation of labeled RNA. Target samples were labeled with Cy5 and Cy3 was used for controls. The input of total RNA in each reaction was 100 ng. After overnight hybridization in an oven (17 h, 65°C, rotation speed 10 rpm), arrays were washed with Gene Expression Wash Buffers 1 and 2 and scanned with a GenePix 4100A (Molecular Devices, Sunnyvale, CA USA). GenePix Pro 6.0 was used for spot to grid alignment, assessment of spot quality, feature extraction and quantification. Subsequent data analyses were performed with STARS. After filtration of low quality spots flagged by GenePix, Lowess normalization of log_2_-expression ratios was performed. The regulated genes were selected by expression difference between the time-points (day 7 was set as a reference) and the cell cultures, aSACs and aSOCs (p<0.05 and expression ratio >2-fold).
